# Long term improvement of knee osteoarthritis after injection of single high/very high volume of very pure PRP: A retrospective analysis of patients optimally managed in dedicated centers

**DOI:** 10.1016/j.reth.2023.12.006

**Published:** 2024-01-03

**Authors:** Didier Prost, Thomas Bardot, Alexandre Baud, Anthony Calvo, Stephane Aumont, Herve Collado, Julien Borne, Olivier Rajon, Antoine Ponsot, Alexandra Malaterre, Yannis Dahak, Guy Magalon, Florence Sabatier, Jeremy Magalon

**Affiliations:** a Regenerative Medicine Department of Excellence, Marseille, France; b Regenerative Medicine Department of Excellence, Lyon, France; c Cell Therapy Laboratory, Hôpital de la Conception, AP-HM, INSERM CIC BT 1409, Marseille, France; d Aix Marseille Univ, INSERM, INRA, C2VN, Marseille, France

**Keywords:** PRP, Knee osteoarthritis, Quality control, Real world evidence

## Abstract

**Introduction:**

PRP is gaining increasing interest for pain relief and improvement of joint function in patients with knee osteoarthritis (KOA) but practices and results remain heterogeneous limiting its adoption as standard of care. Current international recommendations are to collect real-life evidence of efficacy with a systematic monitoring of PRP quality and patients' outcomes. We aimed to analyze the response of patients presenting KOA and treated with standardized PRP injection in routine care. We also investigated the potential contributing factors including patient's phenotype and PRP characteristics.

**Methods:**

Patients with symptomatic KOA and that failed first-line therapy received a single injection of a qualified PRP prepared using medical devices allowing to recover a high/very high volume of very pure PRP. Visual analogue scale (VAS) and Western Ontario and McMaster Osteoarthritis Index (WOMAC) score were recorded at baseline and during 18 months follow-up.

**Results:**

431 patients had available follow-up data at 3 months, 291 at 6 months, 137 at 12 months and 44 at 18 months. PRP induced a significant decrease of WOMAC score at all follow up endpoints (29.2 ± 19.2 at 3 months, p < 0.001 and 25.9 ± 19.7 at 12 months, p < 0.01, compared to 39.7 ± 18.9 at baseline). Similar results were observed for pain VAS (38.9 ± 23.3 at 3 months, p < 0.001 and 35.3 ± 24.1 at 12 months, p < 0.05, compared to 56.0 ± 20.7 at baseline). Changes at 12 months were correlated to baseline scores and to the level of improvement at 3 months. The proportion of OMERACT OARSI responders reached 56.2 % for the total cohort and 60.4 % for severe patients at 6 months. Treatment failure occurred for 8.4 % of patients. Age, BMI or Kellgren–Lawrence grade did not impact on efficacy.

**Conclusion:**

This real-life study evidences the clinical benefit of a standardized high or very high-volume injection of very pure PRP in patients with KOA, including those with a severe grade. It opens perspectives in the positioning of such strategy to delay arthroplasty and provide insights on factors able to anticipate long term efficacy.

## Highlights

• A single injection of a high/very high volume of very pure and biologically controlled PRP allows for a significant improvement in function and pain over 18 months.
• The proportion of responders according to OMERACT-OARSI criteria at 6 months reached 56.2 % and 60.4 % for the total cohort and severe patients, respectively.
• Previous PRP injections, lower baseline WOMAC score or Pain VAS, and patellofemoral localization were criteria associated with poor improvement at 12 months.
• PRP preparation in dedicated centers allows for systematic monitoring of biological qualityand collection of efficacy data in real life at a reasonable cost while strictly adhering to recent international recommendations in the field.

## Abbreviations

AAOS: American Academy of Orthopedic Surgeons
ACD-A: Anticoagulant Citrate Dextrose Solution A
ACR: American College of Rheumatology
AMSSM: American Medical Society for Sports Medicine
BMI: Body Mass Index
CE: Conformité Européenne
CNIL: Commission Nationale de l’Informatique et des Libertés
CTC: Corticosteriods
FDA: Food and Drug Administration
FT: Femorotibial
GFs: Growth Factors
HA: Hyaluronic Acid
IA: Intra-articular
JKOM: Japanese Knee Osteoarthritis Measure
KL: Kellgren Laurence
KOA: Knee Osteoarthritis
MIBO: Minimum Information for studies evaluating Biologics in Orthopaedics
MSC: Mesenchymal Stem Cells
NA: Not Available
OA: Osteoarthritis
OMERACT-OARSI: Outcome Measures in Rheumatology, Osteoarthritis Research Society International
ORBIT: ORthoBIologics InitiaTive
PF: Patellofemoral
PROMs: Patient Reported Outcome Measures
PRP: Platelet-rich plasma
RBCs: Red Blood Cells
RWE: Real World Evidence
RWO: Real World Outcomes
SD: Standard Deviation
VAS: Visual Analogue Scale
WOMAC: Western Ontario and McMaster Osteoarthritis Index

## 1 Introduction

Osteoarthritis (OA) is a degenerative pathology of the cartilage associated to structural and functional changes in the joint [1]. 303 million people are suffering from OA worldwide and its incidence is increasing, causing a substantial burden for the healthcare system. Knee osteoarthritis (KOA) represents approximately 89 % of the burden of OA worldwide [1]. Its diagnostic is confirmed based on a clinical examination seeking mainly for pain and swelling of the knee, and on radiographic features [2]. Available conservative treatments are effective but with limitations. Non-pharmacological approaches, such as dietary supplements, muscle strengthening exercises, are often associated with poor compliance [3]. Pharmacological therapies, including analgesics, non-steroid anti-inflammatory drugs and corticosteroid or hyaluronic acid (HA) injections, provide only temporary benefits sometimes associated with side effects [[4], [5], [6]]. This situation has led to the emergence of injectable “biologic” medication, among which platelet-rich plasma (PRP) is experiencing increasing clinical use. PRP is defined as an autologous plasma suspension of platelets characterized by a higher platelet concentration than peripheral blood [7]. Once locally injected, platelets are activated by physiological activators (collagen, calcium) and release high levels of growth factors (GFs) involved in reparative processes [8]. These GFs act at various levels to restore the joint homeostasis, as reported by pre-clinical models describing chondrocytes anabolism and chondral remodeling, increased HA secretion and down-regulation of inflammation and apoptotic pathways following PRP injections [9,10]. Overall, animal studies described clinical effects in 80 % and disease-modifying effects in 68 % of the studies with attenuation of cartilage damage progression, reduction of synovial inflammation and change in biomarker levels [11]. Ten recent meta-analysis [[12], [13], [14], [15], [16], [17], [18], [19], [20], [21]] have supported the safety and clinical benefit of intra-articular injection of PRP in patients with KOA, of which 8, gathering data from 37 studies, reported a significant improvement in favor of PRP compared to HA concerning pain relief and knee joint function. Consistently, the ESSKA ORBIT consensus group recently recommended the use of PRP in KOA (grade A) over corticosteroids (grade A) and hyaluronic acid (grade B) using a Delphi methodology. Despite popularity and massive use of PRP, medical practices remain heterogeneous limiting its adoption as standard of care [22]. One of the main weaknesses is the lack of a precise biological characterization of the PRP injected as underlined by Chahla et al. [23]. Indeed, substantial differences in the composition of PRP exist according to the PRP preparation protocols, including manual methods or the use of the numerous medical devices currently available [24,25], and their impact on clinical results is a hot topic [26,27]. Thus, performing a biological characterization of the PRP is highly recommended by the International Society for Thrombosis and Haemostasis (ISTH) [28] and the American Academy of Orthopedic Surgeons (AAOS) [29,30]. The AAOS also underlines the importance to set-up registries for post-marketed monitoring of PRP injection. The American Medical Society for Sports Medicine (AMSSM) also pointed out the necessity to realize a systematic biological quality control and promote the collection of Patient-Reported Outcome Measures (PROMs) to assess real-world evidence (RWE) and outcomes (RWO) on regenerative medicine treatments [31]. Although implementation of these recommendations is essential to find the right place of PRP in a broad range of diseases including musculoskeletal field, this is challenging in daily practice, time-consuming and less profitable for physicians. This context led to the emergence of dedicated centers bringing together physicians taking care of patients with trained staffs as well as the necessary equipment and expertise for biological quality control, and the organization of real-life data collection.

Our hypothesis is that the implementation of routine care PRP therapy that fully complies with the recent international recommendations can provide a better appraisal of the early and lasting clinical benefit of PRP injection. We therefore analyzed the response of patients presenting with KOA and treated with a single PRP injection in dedicated centers, and investigate the potential contributing effect of patients’ phenotype, KOA characteristics and PRP composition.

## 2 Materials and methods

### 2.1 Study design

This retrospective observational study gathered data from patients treated for KOA by different physicians in two dedicated centers specialized in PRP injections (Remedex, Marseille & Lyon, France) which operate according to similar standardized procedures. Data were routinely recorded in the medical files between January 2021 and December 2022. Patients included males and females ≥18 years of age with symptomatic KOA meeting the American College of Rheumatology (ACR) criteria [32] and failed usual first-line therapy (physical therapy and oral non-steroidal anti-inflammatory drugs). All patients included received a PRP injection in only one knee and had both WOMAC and Pain VAS results available at initiation and the first evaluation at 3-month follow-up. All patients provided informed consent for injection and collection/utilization of their data in the context of routine care. All procedures were performed after conformity declaration to the French National Authority for data privacy (CNIL, Commission Nationale de l’Informatique et des Libertés).

### 2.2 PRP preparation

PRP was prepared using either Hy Tissue Tube 20 or 50 devices (Fidia, Abano Terme, Italy) based on physicians’ habits. Accordingly, either 18 or 45 mL of blood was collected by venipuncture using a 21-gauge needle filling one 20 or 50 mL syringe containing 2 or 5 mL of ACD-A (Fidia, Abano Terme, Italy). The blood was transferred into the corresponding Hy-tissue device (Fidia, Abano Terme, Italy) and centrifuged using the Duografter II equipment during 10 min. All plasma was recovered to obtain approximatively 10 or 20 mL of PRP through the Push-out system with Hy Tissue Tube 20 or 50 devices, respectively. 300 μL of whole blood and PRP preparation were sampled to determine platelets, leukocytes and RBCs concentrations. Following PRP production, release quality criteria were checked to ensure the production of a very pure PRP corresponding to a platelets purity ≥90 % [33] with limited loss of platelets defined by a recovery rate ≥50 %.

### 2.3 Biological characterization of PRP

Platelets, leukocytes and red blood cells concentrations were determined using automated haematology blood cell analyzers Micros ES-60 (Horiba, Montpellier, France) in accordance with published guidelines [34].

### 2.4 Injection

The intra articular knee injection was performed without local anesthesia using a 21-gauge needle after conventional skin aseptic decontamination. The injections were performed under using anatomic landmark palpation-guided approach (n = 411), ultrasound guidance (n = 7) or scopic guidance with arthrography (n = 13).

### 2.5 Evaluation tools and follow-up

All data were collected using Remedex Follow-Up which is an electronic medical records tool. Demographic data including age, sex, body mass index (BMI) as well as previous IA injection and location/severity of KOA using Kellgren Laurence (KL) radiological scale were assessed before the PRP injection. The patient's subjective symptoms were assessed using the Western Ontario and McMaster Universities Arthritis Index (WOMAC) score, which is a patient-reported outcome measure for the assessment of lower limb osteoarthritis [35] ranging from 0 (no pain) to 100 (worse pain). Knee pain was evaluated on a visual analogue scale (VAS) from 0 (no pain) to 10 (maximal pain). These parameters were evaluated before the injection and 3 months (M3) after the procedure. Patients were also asked to fill regularly these questionnaires using the electronic tool at 6 (M6), 12 (M12) and 18 months (M18). Patients were considered as severe if they presented a functional impairment ≥40 on WOMAC score, a pain VAS ≥40 and a grade 3 or 4 knee OA according KL classification, supported by data from knee arthroplasty registry [36]. Patients who did not answer the questionnaire one month after the theorical schedule were classified as lost to follow-up, whereas patients with ongoing follow-up were classified as follow-up in progress.

### 2.6 Prediction of factors associated to PRP responses

Three categories of factors with potential value to anticipate the clinical impact of PRP injection were considered: patients characteristics (age, sex, BMI, initial and change in WOMAC score and pain VAS at M3), KOA localization/severity, and PRP preparation (Hy Tissue Tube 20 or 50). These factors were analyzed for their possible association with treatment failure, responder's status and change in WOMAC or pain VAS defined at M12 by reference to baseline. Patients were classified as responders if they met the OMERACT-OARSI criteria as follows [37]: i) high improvement in pain or function ≥50 % and absolute change ≥20 or ii) improvement ≥20 % and absolute change ≥10 in at least 2 of the 3 following criteria: pain, function and patient's global assessment. Treatment failure was defined as the realization of arthroscopy or knee arthroplasty on the treated joint during the follow-up.

### 2.7 Statistical analysis

Statistical analysis was performed with EasyMedStat (version 3.23) and GraphPad Prism v9.0.2 software (San Diego, CA, USA) for graphical representation of figures. The normality of the distribution of quantitative parameters was assessed by conducting the Kolmogorov–Smirnov test. Continuous variables were presented as mean (SD) or median (interquartile range) for normally and non-normally distributed data. A univariate analysis was carried out by using the Student t-test to find significant differences between two groups in normally distributed parameters, while the Mann–Whitney U test was performed in non-normally distributed variables. Discrete variables were presented as numbers and percentages and compared with chi-squared or Fisher's exact test. The Kruskal–Wallis test was used to analyze outcome variables according to the different modalities of categorial variables. Spearman's correlation was used to assess linear dependence between two continuous variables. Repeated-measures analyses were performed with Friedman's test. If the null hypothesis of Friedman's test was rejected, post-hoc pairwise analyses were performed with Nemenyi's test. Alpha risk was set to 5 % (α = 0.05). Multivariate analysis using logistic regression models were performed to determine potential predictive factors that may be independently associated to PRP response at M12 after adjustment on controlled variables. The relevant variables were selected from the bivariate analysis, based on a threshold p value ≤ 0.2. Controlled variables selected on their clinical interest were age, BMI, and KL radiological score. The final model expressed the odds ratios and 95 % confidence intervals. All tests were two-sided. Statistical significance was defined as p < 0.05.

## 3 Results

### 3.1 Characteristics of patients and follow-up

**Fig. 1:**
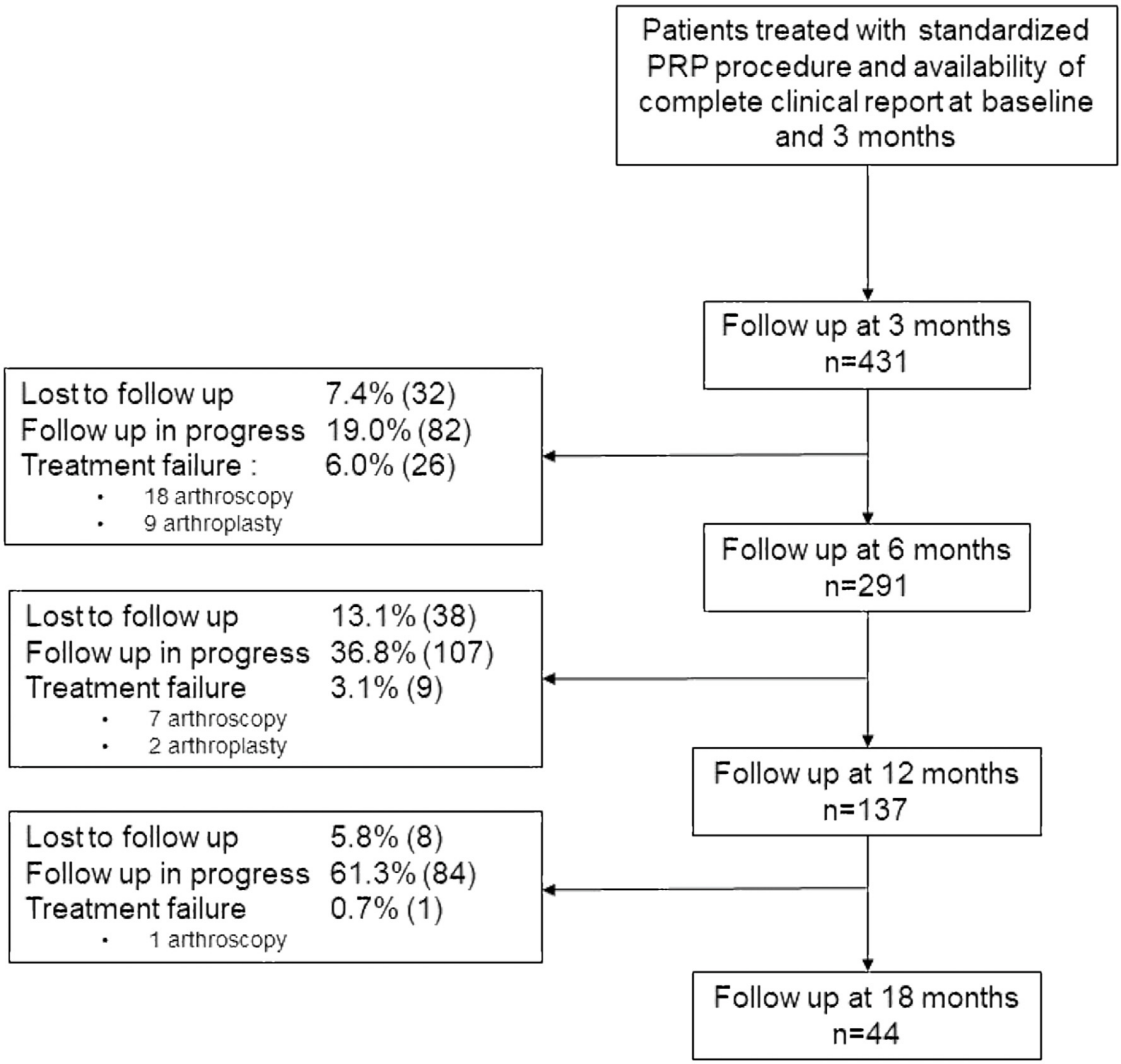
Participant flowchart.

Data from 431 patients (50.5 % male) were collected. Full characteristics of the patients treated are described in Table 1 whereas baseline characteristics at M6, M12 and M18 are available in Supplemental Table 1. Age was most frequently ranging from 60 to 79 years (50.6 %) followed by patients aged from 40 to 59 years (36.6 %). BMI was mainly comprised between 25 and 30 kg/m2 (41.1 % of the patients) whereas obese patients (BMI >30 kg/m2) represent 17.7 % of the cohort. The radiological KL grade of KOA was grade 2 (36.4 %) followed by grade 3 (28.5 %), 4 (18.8 %) and 1 (13.3 %), with an isolated femorotibial or patellofemoral (PF) location for 68.8 % and 11.8 % of the patients respectively. 226 patients (52.4 %) previously received an IA injection. From the 431 patients, 124 (28.8 %) were considered severe and potentially candidate to knee arthroplasty. Demographic characteristics and prevalence of co-morbidity factors in this subgroup of severe patients did not significantly differ compared to the entire cohort. All patients had available follow-up data at M3, 291 at M6, 137 at M12 and 44 at M18. During the 18-month observation period, 40 patients (9.3 %) received an additional local injection after the PRP therapy, including 4 patients who received HA, 2 patients who received corticosteroids and 34 patients who received PRP. The full rate of patients lost to follow-up, follow-up in progress or treatment failure at each period is detailed in Fig. 1.

**Table 1:** Baseline Characteristics of patients (n = 431).

**Men**	50.5 (218)	**KL grade**	
**Age (y)**		*1*	13.3 (57)
*18–39*	4.4 (19)	*2*	36.4 (157)
*40–59*	36.6 (158)	*3*	28.5 (123)
*60–79*	50.6 (218)	*4*	18.8 (81)
*≥80*	8.4 (36)	*NA*	3.0 (13)
**BMI (kg/m**^**2**^**)**		**Localization**	
*<25*	36.0 (155)	*Femorotibial*	68.8 (296)
*25–30*	41.1 (177)	*Patellofemoral*	11.8 (51)
*>30*	17.6 (76)	*FT + PF*	16.7 (72)
*NA*	5.3 (23)	*NA*	2.7 (12)
**Previous treatment**		**Sport practice**	
*None*	46.4 (200)	*None*	33.9 (146)
*HA*	29.0 (125)	*Active*	64.0 (276)
*CTC*	10.9 (47)	*NA*	2.1 (9)
*PRP*	10.0 (43)	**Global health**	
*≥ 2*	2.6 (11)	*Poor*	12.3 (53)
*NA*	1.1 (5)	*Moderate*	25.1 (108)
		*Excellent*	24.3 (105)
		*NA*	38.3 (165)

Data represent frequency (number of patients); y: year; BMI: Body Mass Index; HA: hyaluronic acid; CTC: corticosteroids; PRP: platelets rich plasma; KL: Kellgren Laurence; FT: femorotibial; PF: patellofemoral; NA: not available.

### 3.2 Biological characteristics of injected PRP

Hy Tissue Tube 20 was used in 230 procedures, whereas Hy Tissue Tube 50 was used in 201 procedures according the final volume desired by the physician. Comparison of the PRP obtained from the two devices revealed an expected significant difference in the volume of injected PRP (8.6 ± 1.2 for Hy Tissue Tube 20 and 17.0 ± 3.1 mL for Hy Tissue Tube 50; p < 0.001) and the number of injected platelets (3.7 ± 1.1 for Hy Tissue Tube 20 and 9.0 ± 2.9 billion for Hy Tissue Tube 50; p < 0.001). Although PRP obtained with both devices were considered very pure [37], Hy Tissue Tube 20 provided a significantly purer PRP compared to Hy Tissue Tube 50 (97.6 ± 1.7 for Hy Tissue Tube 20 and 95.2 ± 2.2 % for Hy Tissue Tube 50; p < 0.001) whereas recovery rate was similar (83.5 ± 13.1 for Hy Tissue Tube 20 and 83.7 ± 12.3 % for Hy Tissue Tube 50; p = 0.72). Conformity of PRP production was achieved for 417 preparations (96.8 %). Detailed biological characterization obtained with each devices is provided in Supplemental Table 2.

### 3.3 Pain during injection

Mean pain VAS during injection was 30.6 ± 27.3 and 117 patients (27.1 %) reported a pain VAS ≥50 during injection. No significant difference was noted between the use of Hy Tissue Tube 20 device (mean pain VAS 31.4 ± 28, 67 patients (29.1 %) with a pain VAS ≥50 during injection) compared to Hy Tissue Tube 50 device (mean pain VAS 29.6 ± 27, 50 patients (24.8 %) with a pain VAS ≥50 during injection, p = 0.47).

### 3.4 Comparison of clinical assessments

**Fig. 2:**
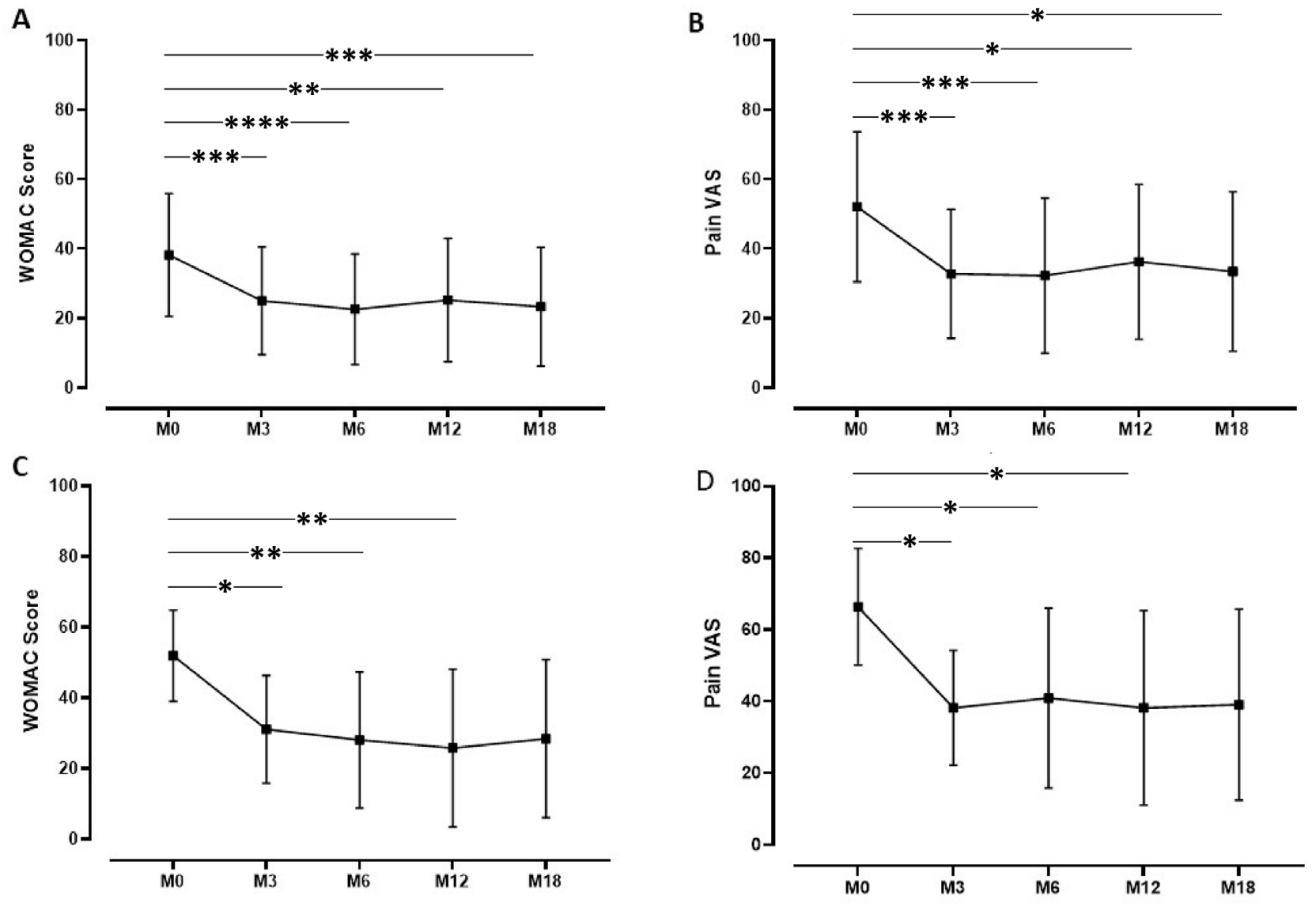
Evolution of WOMAC score and Pain VAS for patients with 18 months follow-up. A,B: all patients (n = 44); C,D: severe patients (n = 11). ∗: p ≤ 0.05, ∗∗: p ≤ 0.01, ∗∗∗: p ≤ 0.001, ∗∗∗∗: p ≤ 0.0001.

**Fig. 3:**
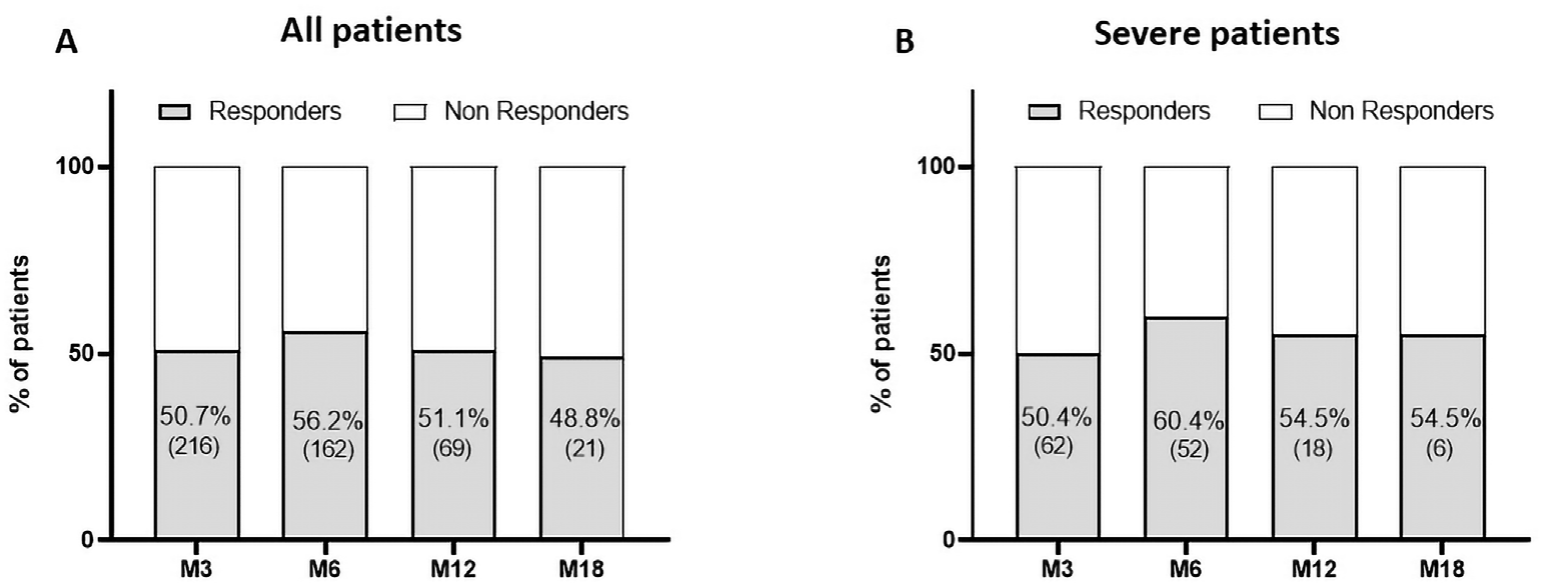
Proportion of OMERACT-OARSI responders among all patients (A) and severe patients (B) at different follow-up (Below the figure): Missing data for 4 patients at M3 (1 severe), 3 patients at M6 (no severe), 2 patients (2 severe) at M12.

PRP injection was effective in improving knee functional status and reducing symptoms, as shown by the significant decrease of both WOMAC score and pain VAS observed at all follow-up endpoints, until M18, compared to baseline (Fig. 2 A,B and Table 2A). The mean change from baseline to M12 (137 patients) were −11.3 ± 16.7 and −18.6 ± 25.1 for WOMAC score and pain VAS, respectively. Approximatively 70–80 % of the patients presented an improvement in WOMAC score and pain VAS during the different follow-up periods achieving a mean percentage of improvement of 51.0 ± 32.1 % for WOMAC score and 57.2 ± 49.9 for pain VAS at M12. When restricted to severe patients potentially candidates to knee arthroplasty, similar changes in outcomes were observed (Fig. 2C and D and Table 2B). The highest proportion of OMERACT OARSI responders was reached at M6 (56.2 % for the total cohort and 60.4 % for severe patients) with a slight decrease at M12 and M18 (Fig. 3). Treatment failure occurred for 36 patients (8.4 %) among which 7 were identified from the severe ones (5.6 %). No adverse events were reported. 78 patients (18.1 %) were lost to follow-up especially between M6 to M12 (38 patients). Among them, 47 (60.2 %) presented an improvement in WOMAC score at the last follow-up they filled.

**Table 2A:** Clinical outcomes for the global population.

	M0	M3	M6	M12	M18
**Number of patients**	431	431	291	137	44
**WOMAC Score**	39.7 ± 18.9	29.2 ± 19.2	28.5 ± 20.1	25.9 ± 19.7	23.4 ± 17.1
Change in WOMAC score	–	−10.5 ± 16.0	−11.1 ± 17.3	−11.3 ± 16.7	−14.9 ± 21
Proportion (n) of patients improved	–	73.8 (318)	74.6 (217)	73.7 (101)	72.7 (32)
% improvement (improved patients)	–	43.6 ± 26.3	48.2 ± 28.7	51.0 ± 32.1	52.4 ± 32.3
% improvement (total patients)	–	18.4 ± 78.0	18.3 ± 118	28.2 ± 51.7	32.6 ± 43.5
**Pain VAS**	56.0 ± 20.7	38.9 ± 23.3	38.2 ± 24.6	35.3 ± 24.1	33.5 ± 22.9
Change in pain VAS	–	−17.2 ± 23.2	−18.7 ± 24.1	−18.6 ± 25.1	−18.6 ± 29.6
Proportion (n) of patients improved	–	68.9 (297)	69.8 (203)	69.3 (95)	65.9 (29)
% improvement (improved patients)	–	50.2 ± 26.2	53.8 ± 25.5	57.2 ± 49.9	54.5 ± 29.5
% improvement (total patients)	–	25.2 ± 62	27.4 ± 69.0	27.9 ± 61.9	22.2 ± 61

Data represent mean ± standard deviation except for proportion of patients improved represented as frequency (number of patients).

**Table 2B:** Clinical outcomes restricted to severe patients.

	M0	M3	M6	M12	M18
**Number of patients**	124	124	86	35	11
**WOMAC Score**	55.3 ± 11.4	40.7 ± 16.9	39.9 ± 17.7	35.5 ± 22.0	28.4 ± 22.4
Change in WOMAC score	–	−14.6 ± 15.8	−15.2 ± 16.7	−16.9 ± 21.7	−23.4 ± 29.0
Proportion (n) of patients improved	–	80.6 (100)	77.9 (67)	74.3 (26)	81.8 (9)
% improvement (improved patients)	–	35.7 ± 21.9	39.4 ± 21.8	48.6 ± 33.4	55.6 ± 35.6
% improvement (total patients)	–	25.9 ± 28.3	27.4 ± 30.2	32.6 ± 39.9	41.3 ± 45.0
**Pain VAS**	69.4 ± 13.9	50.9 ± 21.7	49.1 ± 23.2	45.1 ± 26.8	39.1 ± 26.6
Change in pain VAS	–	−18.5 ± 23.4	−22.7 ± 23.5	−25.8 ± 25.9	−27.3 ± 34.3
Proportion (n) of patients improved	–	70.2 (87)	75.6 (65)	74.3 (26)	81.8 (8)
% improvement (improved patients)	–	41.3 ± 23.1	44.8 ± 22.3	49.9 ± 28.9	58.6 ± 31.1
% improvement (total patients)	–	24.6 ± 35.8	30.7 ± 32.3	36.7 ± 36.9	35.8 ± 47.1

Data represent mean ± standard deviation except for proportion of patients improved represent as frequency (number of patients).

### 3.5 Factors associated to PRP responses

**Fig. 4:**
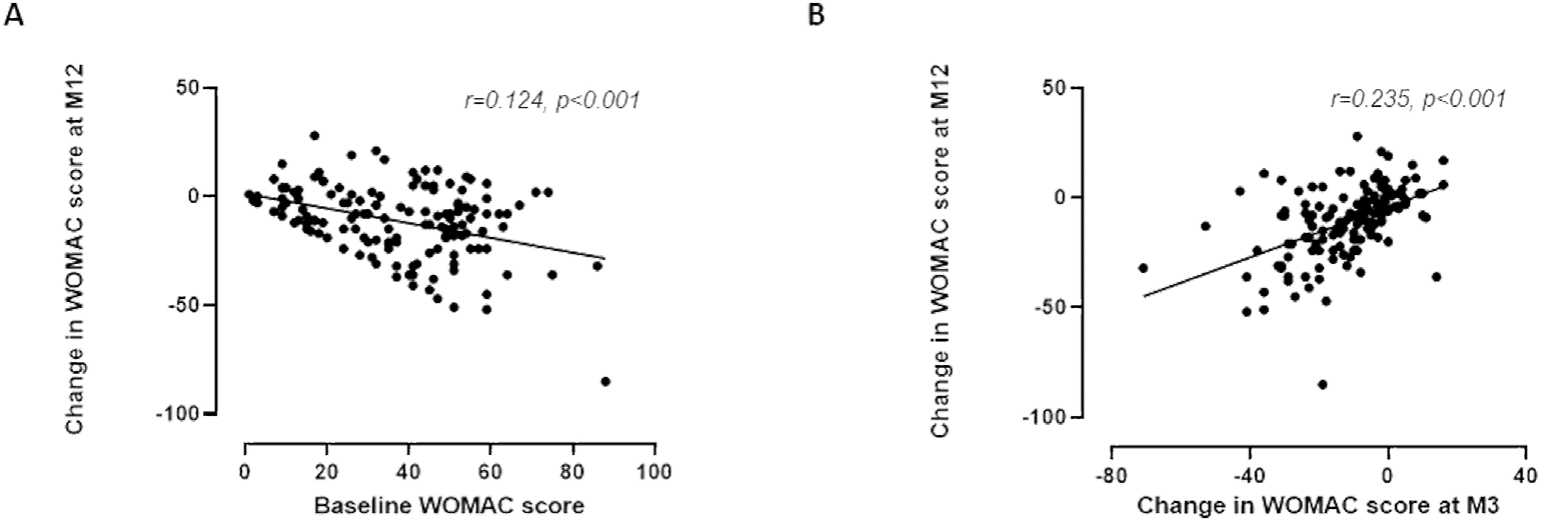
Correlation between change in WOMAC score at M12 and baseline WOMAC Score (A) or change in WOMAC score at M3 (B).

By univariate analysis comparing patients according to treatment failure or not, only change in WOMAC score and pain VAS at M3 were significantly different between groups (−1.89 ± 16.3 and −8.61 ± 24 versus −11.27 ± 16.6 and −18.0 ± 2.31, p = 0,001 and p = 0,026, respectively), supporting that a lower improvement at early endpoints is associated to treatment failure. When non-response at M12 according to the OMERACT OARSI criteria was considered as outcome variable, similar differences were observed between non-responders and responders (change in WOMAC score and change in pain VAS at M3: −8.2 ± 14.0 and −10.6 ± 22.4 versus −16.7 ± 13.0 and −30.0 ± 20.6; p < 0.001 and p < 0.001 respectively). Non-responder patients also had lower pain VAS at baseline (48.6 ± 23.7) compared to responders (58.9 ± 19, p = 0.018). Changes in WOMAC score and pain VAS at M12 were also found to be correlated with changes observed at M3 (r = 0.235, p < 0.001 and r = 0.343, p < 0.001 respectively) and with corresponding baseline values (r = 0.124, p < 0.001 and r = 0.231 p < 0.001, respectively; Fig. 4A and B). Collectively, these data indicate that patients with marked symptoms at baseline may benefit the most from the high/very high volume of very pure PRP injection, and that the reduction of symptoms at M3 makes it possible to anticipate a persistent improvement over time.

**Fig. 5:**
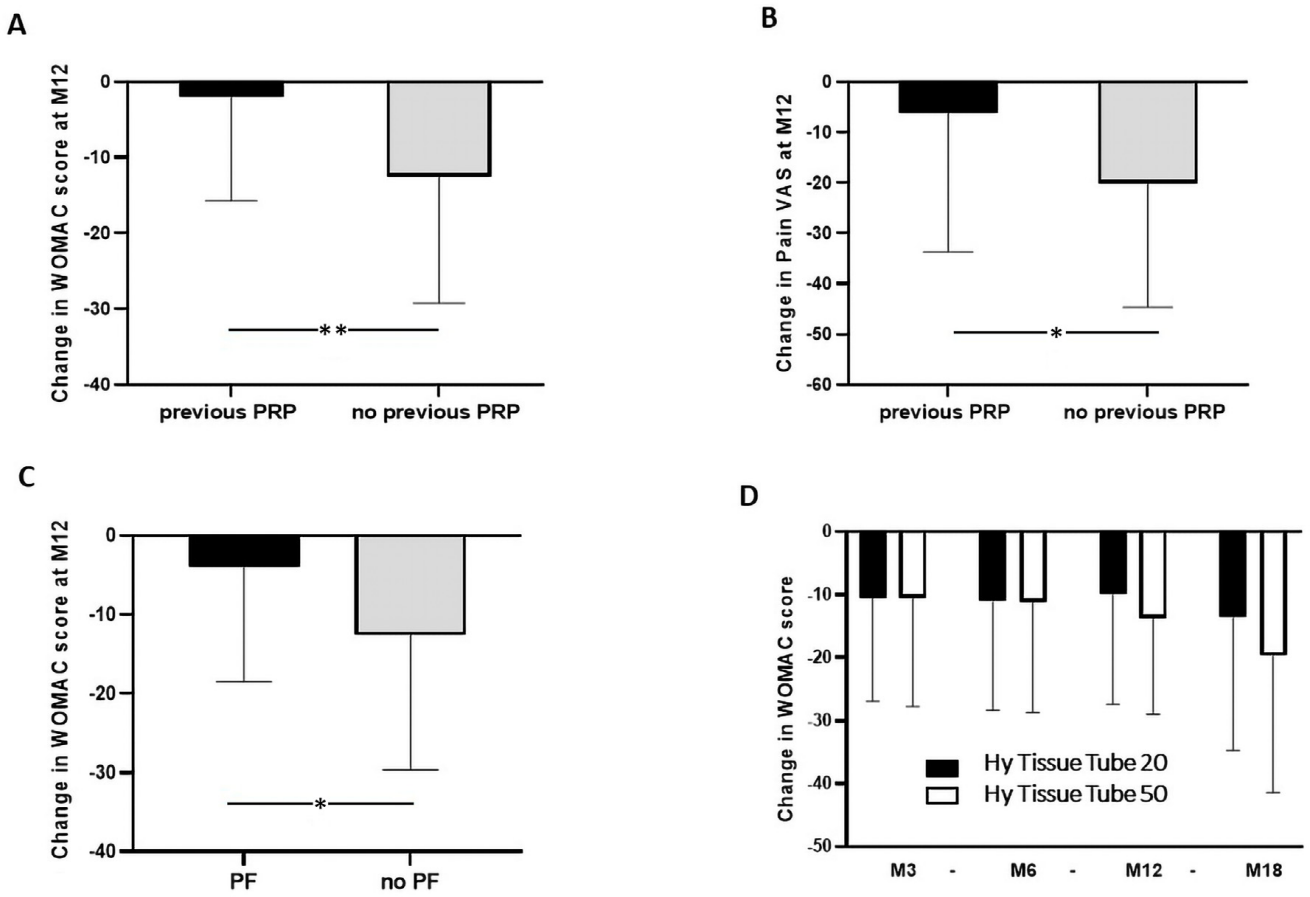
Univariate analysis reporting the impact of previous PRP injection on WOMAC (A) and Pain VAS (B) change at M12, PF KOA on WOMAC change at M12 (C) and PRP device on WOMAC change during follow-up (D). PF: patellofemoral; ∗: p ≤ 0.05, ∗∗: p ≤ 0.01.

Consistently, we also observed that among non-responder patients at M12, the frequency of a previous PRP injection was significantly higher compared to responders (15.1 % vs 4.3 %, p = 0.029). Similarly, the extent of improvement in WOMAC score and pain VAS at M12 was significantly higher in patients who had not received PRP injection compared to patients previously treated (−12.5 ± 16.7 and −20.1 ± 24.6 vs −2.0 ± 13.7 and −6.1 ± 27.5, p = 0.022 and 0.049, respectively; Fig. 5A and B).

Regarding baseline characteristics of patients, age, BMI and KL grade did not significantly differ between responders and non-responders, and did not significantly associate with change in WOMAC score and pain VAS evaluated at M12. However, we found that the decrease in WOMAC score at M12 was significantly less pronounced in patients with an isolated PF KOA compared to patients with other localization (−4 ± 14.5 vs −12.6 ± 17, p = 0.015; Fig. 5C). The association of this KOA characteristic with the change in pain VAS or response to PRP according to OMERACT OARSI criteria revealed a trend without reaching statistical significance (p = 0.2).

Regarding the modality of PRP preparation, a trend was identified revealing higher improvement in WOMAC score change at M12 and M18 when using Hy Tissue Tube 50 device, corresponding to significantly higher volume and injected number of platelets, compared to using Hy Tissue Tube 20 (−13.8 ± 15.2 and −19.7 ± 21.7 versus −9.9 ± 17.4 and −13.6 ± 21.1, p = 0.18 and p = 0.40 respectively; Fig. 5D). Deeper analysis of patients’ characteristics treated with each device did not reveal any difference regarding age and BMI or initial WOMAC score and pain VAS (Supplemental Tables 3 to 6). However, patients treated with Hy Tissue Tube 50 had higher proportion of patients with severe KL grade (p = 0.007) and lower proportion presenting isolated PF knee OA (p = 0.046) and previous HA treatment (p = 0.001).

Finally, multivariate analysis (Table 3) confirmed that the absence of previous PRP injection and the change in pain VAS at M3 were the only independent predictors of response to PRP defined according to OMERACT OARSI criteria at one-year post injection. When considering change in WOMAC score at M12, characteristics such as previous PRP treatment, lower baseline score and isolated PF independently associated with lower functional improvement. Similar predictive factors were identified for pain VAS at M12 excepted isolated patellofemoral localization. The modality of PRP preparation using Hy Tissue Tube 50 could not identify patients who are more likely to be improved.

**Table 3:** Multivariable analysis of factors associated with PRP therapy outcomes.

Variable	Modality	Odds Ratio [95 % CI]	*p value*
**Response to PRP (OMERACT OARSI criteria at M12)**			
Age	≥60	0.939 [0.378; 2.33]	*0.89*
IMC	25–30	1.46 [0.581; 3.68]	*0.42*
	>30	0.364 [0.0964; 1.38]	*0.14*
KL score	2	1.68 [0.499; 5.67]	*0.40*
	3	1.76 [0.444; 7.02]	*0.42*
	4	1.26 [0.27; 5.86]	*0.77*
Patellofemoral KOA	yes	0.668 [0.184; 2.42]	*0.54*
Previous PRP	yes	0.151 [0.0251; 0.911]	***0.0392***
Change WOMAC M3	–	1 [0.961; 1.04]	*0.99*
Baseline Pain VAS		1.01 [0.985; 1.03]	*0.45*
Change Pain VAS M3		0.959 [0.932; 0.986]	***0.00308***

**Change in WOMAC score at M12**			
Age	≥60	2.82 [-2.3; 7.93]	*0.28*
IMC	25–30	0.942 [-4.54; 6.42]	*0.73*
	>30	3.57 [-6.28; 13.42]	*0.47*
KL score	2	−2.27 [-9.86; 5.33]	*0.55*
	3	−7.4 [-15.53; 0.732]	*0.07*
	4	−2.88 [-11.89; 6.12]	*0.52*
Patellofemoral KOA	yes	8.65 [1.19; 16.12]	***0.0235***
Previous PRP	yes	11.96 [5.11; 18.82]	***<0.0001***
Baseline WOMAC		0.4 [-0.571; −0.229]	***<0.0001***
Hy Tissue Tube 50		−1.94 [-7.65; 3.77]	*0.50*

**Change in Pain VAS at M12**			
Age	≥60	−6.01 [-14.28; 2.26]	*0.15*
IMC	25–30	1.2 [-7.27; 9.68]	*0.78*
	>30	10.36 [-1.47; 22.18]	*0.085*
KL score	2	−1.1 [-12.84; 10.65]	*0.85*
	3	−8.83 [-21.31; 3.65]	*0.16*
	4	−0.0931 [-14.31; 14.12]	*0.99*
Patellofemoral KOA	yes	9.02 [-2.05; 20.09]	*0.11*
Previous PRP	yes	19.12 [6.08; 32.16]	***0.0044***
Baseline Pain VAS		−0.644 [-0.825; −0.463]	***<0.0001***
Hy Tissue Tube 50	yes	−2.08 [-10.35; 6.19]	*0.62*

## 4 Discussion

To our knowledge, this study is the first to report in a comprehensive manner the outcomes of 431 patients with KOA optimally managed regarding the characterization of the injected PRP and the collection of data in a real-life setting. A single injection of high or very high volume of a very pure PRP led to a high proportion of responders with a significant improvement in WOMAC score and pain VAS until 18 months. Importantly the long-term benefit of PRP was also objectified in severe patients, independently of patient's comorbidities and disease phenotype.

Of note, our cohort benefited from a very homogeneous PRP quality that complied with stringent specifications ensuring a very high purity and high dose of platelets. Such standardization, is expected to contribute to the robustness of the results. Only 8.4 % of the cohort had a treatment failure whereas more than 50 % of the patients were considered as responders at 6 months. Although the choice of the OMERACT OARSI–based approach to qualify responders was not advantageous in our case, as we could not collect data on a patient global assessment, we considered this composite score as the most stringent definition of response, also allowing a broad comparison with recent real-life studies. Chopin et al. [38] described a responder's rate of 43.1 % at 7 months following two low volume injections one month apart, whereas Saita et al. [39] reached 62.1 % at 12 months using three 4–5 mL injections decreasing to 50.9 % for patients presenting KL grade 4 knee OA. This opens the debate about the necessity to repeat small volume injections rather than targeting a single large volume injection for KOA. Indeed, although the initial use of PRP by Marx in regenerative field described high volume and high concentration of platelets [40], the emergence of commercial medical devices to prepare PRP had modeled the procedure use with hyaluronic acid protocols of injection i.e. low volume and repeated injections and were poorly interested on characteristics of PRP injected. However, high-volume injection [41] of very pure PRP [42], targeting high dose of platelets [43] and taking into consideration the large capacity of the knee joint [44] was recently described with interesting success rates. In line with this, our study suggests that an increased volume and dose of platelets is safe and could led to a prolonged benefit over time, as evidenced by a higher change in WOMAC score at 18 months when Hy Tissue Tube 50 device was used. The determinant contribution of Hy Tissue Tube 50 to efficacy could not be statistically demonstrated, but results deserve to be more extensively assessed in dedicated analysis with a higher number of patients with long term follow-up. However, we strongly believe that, more sophisticated surrogate markers of PRP bioactivity are needed to anticipate PRP-associated clinical efficacy, as recently suggested [[45], [46], [47]].

One of the most striking findings of this study are the results obtained in patients defined as severe. Referring to the patients described in the Dutch University Hospital Registry for knee arthroplasty [36] our severe patients could have been candidate for knee arthroplasty, as they similarly presented important functional impairment and pain and were restricted to grade 3 or 4 knee OA according to KL radiological score. Among them, only 5.6 % had a treatment failure, whereas 60.4 % and 54.5 % were responders at 6 months and 12 months. Consistently, the retrospective work from Sanchez [48] showed that 85.7 % of patients receiving regular 8 mL PRP injections did not undergo knee arthroplasty after five-year follow-up. These findings clearly raise the questions about the place of PRP injections as an alternative or a potential strategy to postpone knee arthroplasty, where recent data indicated a higher risk of failure in middle-age patients compared to older [49].

One of the interests of this kind of study with large cohort of patients is also to identify factors, related to patient selection and PRP quality, in order to better anticipate long term clinical benefit. Conflicting results were published regarding the impact of age, BMI and KL radiological score on response. In line with our results, Sanchez et al. [50] did not observe difference in the frequency of responders according to BMI or age, and the absence of alteration in the concentration of growth factors within PRP from obese patients has been recently reported [51]. Although consensus exists favoring the use of PRP in early KOA based on the poorer clinical response of patients with increased KL grade [39], our study did not demonstrate such association. Our choice to use high volume and very pure PRP could partly account for the satisfactory outcomes in severe patients. Moreover, a recent meta-analysis indicated that the degree of cartilage damage did not impact on PRP efficacy [52]. We also observed that patients presenting a PF KOA are more likely to have lower functional improvement at 12 months. The impact of KOA localization is poorly documented but our results agree with that from ESSKA ORBIT consensus group reporting that magnitude and longevity of improvement in this case remains uncertain. Interestingly, Chopin et al. [38] reported heel-to-buttock distance >35 cm to be associated to poorer response. In our case, the most spectacular findings were the correlation between the improvements of the WOMAC score at 12 and 18 months and a severe initial WOMAC score or a marked change in WOMAC at 3 months. In addition, baseline WOMAC score and Pain VAS appeared as independent predictors of their respective improvement at 12 months. Together with the retrospective analysis of Kikuchi et al. [53] showing that a higher Japanese KO measure score at baseline is a contributing factor of PRP efficacy, our data indicate that patients with pronounced symptoms are those who may benefit the most from PRP therapy. In the same purpose, in our study, previous PRP injection identified patients with a lower rate of response suggesting that it could be rationale to avoid additional PRP procedures in case of absence of functional response at 3 months. From a medico-economic perspective, repetition of PRP injections could be rather reserved to responder patients with reappearance of KOA symptoms with the aim to delay or avoid knee arthroplasty, as recently demonstrated by Sanchez et al. [47]. This raises the question of final price for PRP injection which vary from US$300 to US$600 in our dedicated centers while different studies reported a range from US$500 to US$2500 [54]. In a markov devision analysis and assuming a US$728 cost for PRP injection, Rajan et al. [55] conclude that PRP injections were not cost-effective for delaying the need for knee arthroplasty. In this context, the scientific community faces the challenge of improving the qualification of the injected PRP in daily settings while ensuring affordable cost to enhance both credibility and cost-effectiveness of this therapy. Centralization of PRP preparation in dedicated centers seems to be the best option to control PRP procedure and quality at a reasonable cost, and we share with our colleagues from the Spanish local blood bank that this should be given priority in treatments involving PRP products [56]. This is the reason why we have set up in 2021 a dedicated network of PRP centers in France called Remedex inviting physicians to follow the recent international recommendations in a completely centralized manner including patient calendar management, standardized PRP preparation and quality control and implementation of patients' follow-up data in an authorized registry. Furthermore, the organization that has been set up in these centers include the whole completion of patients’ follow-up to ensure an extremely low rate of lost to follow up and strengthen the value of the data and conclusions obtained. Thus, our study is line with the recent recommendations from scientific societies like the AAOS [29] or the AMSSM [31] that encouraged the post market monitoring of PRP injection combined with extensive description of PRP procedure and biological quality, in order to collect information from routine clinical practice. Such strategy based on real-word evidence provides pertinent supplement to randomized controlled trials which have some limitations in the field of PRP evaluation related to the heterogeneity manufacturing processes, the numerous phenotypes existing to describe KOA, and generally associated to short term follow-up period (6 months to 1 year). In support of, the FDA has stated that they will also consider real-world evidence and associated outcomes when reviewing information on regenerative medicine treatments [57].

The limitations of this study are the absence of randomization and control group which are inherent to real world evidence study and the limited numbers of patients with follow-up at M18. Also, the choice of the PRP preparation devices based on physicians’ habits introduce a selective bias to the study. Data regarding baseline severity of deformity and asymmetry of symptoms between both knees were not collected and could influence clinical results. Finally, this type of study uses Patient-Reported Outcome Measures reflecting an improvement in symptoms without assessing a modification of the disease using imaging tools which are difficult to set up in real life.

The strengths of this study are mainly the high number of patients analyzed following international recommendations. Another important strength to underline is the organization that has been set up to limit the proportion of lost to follow-up which could bias the results. Furthermore, this bias was circumvented by the analysis of the last follow-up filled by this population showing an improvement in WOMAC score in more than 60 % of the patients concerned.

## Ethics approval and consent to participate

All procedures were performed after conformity declaration to the French National Authority for data privacy (CNIL, Commission Nationale de l’Informatique et des Libertés).

## Consent for publication

All patients provided informed consent for injection and collection/utilization of their data in the context of routine care.

## Author contributions

DP, TB, AB, AC, SA, HC, GM, FS, JM contributed to the conceptualization of the work.

DP, TB, AB, AC, SA, HC, JB, OR, AP, AM, YD contributed to the writing – review & editing of the work.

JB, OR, AP contributed to the formal analysis of the work.

AM, YD contributed to the data curation of the work.

AM, YD contributed to the investigation of the work.

GM, FS, JM contributed to the methodology of the work.

GM, FS, JM contributed to the writing – original draft of the work.

## Funding

Not applicable.

## Availability of data and material

The datasets generated and/or analyzed during the current study are not publicly available due to privacy but are available from the corresponding author on reasonable request.

## Declaration of competing interest

The authors of this manuscript declare relationships with the following companies: DP, JB, OR, AP GM, FS, JM have shares in Remedex. JM received honoraria for educational support from Fidia, Horiba, Macopharma, Arthrex and Horus.

These manufacturers had no role in the development of this manuscript or its decision for publication. The other authors declare that they have no competing interest.
