# Supplementary material for: Long term improvement of knee osteoarthritis after injection of single high/very high volume of very pure PRP: A retrospective analysis of patients optimally managed in dedicated centers
Source: Regen Ther. 2024 Jan 3;25:203–12. doi: 10.1016/j.reth.2023.12.006 (PMC10792744; doi:10.1016/j.reth.2023.12.006)
Supplement: Multimedia component 1 [file mmc1.docx]

Table S1. Baseline Characteristics of patients that completed follow-up at 6 months (n=291), 12 months (137) and 18 months (n=44)

|  | 6 months | 12 months | 18 months |
| --- | --- | --- | --- |
| Men | 47.4 (138) | 40.1 (55) | 45.4 (20) |
| Age (y) |  |  |  |
| *18-39* | 3.4 (10) | 2.2 (3) | 4.5 (2) |
| *40-59* | 37.1 (108) | 38.7 (53) | 41.0 (18) |
| *60-79* | 50.6 (147) | 55.5 (76) | 50.0 (22) |
| *≥80* | 8.9 (26) | 3.6 (5) | 4.5 (2) |
| BMI (kg/m²) |  |  |  |
| *<25* | 34.0 (99) | 35.0 (48) | 38.6 (17) |
| *25-30* | 40.5 (118) | 44.5 (61) | 43.2 (19) |
| *>30* | 20.7 (60) | 16.1 (22) | 18.2 (8) |
| *NA* | 4.8 (14) | 4.4 (6) | 0 (0) |
| Previous treatment |  |  |  |
| *None* | 47.4 (138) | 52.6 (72) | 59.1 (26) |
| *HA* | 30.2 (88) | 24.1 (33) | 18.2 (8) |
| *CTC* | 11 (32) | 12.4 (17) | 13.6 (6) |
| *PRP* | 8.6 (25) | 10.2 (14) | 9.1 (4) |
| *≥ 2* | 1.4 (4) | 0 (0) | 0 (0) |
| *NA* | 1.4 (4) | 0.7 (1) | 0 (0) |
| KL grade |  |  |  |
| *1* | 13.0 (38) | 16.0 (22) | 18.2 (8) |
| *2* | 34.0 (99) | 35.8 (49) | 38.6 (17) |
| *3* | 29.2 (85) | 27.7 (38) | 25.0 (11) |
| *4* | 20 (58) | 16.1 (22) | 15.9 (7) |
| *NA* | 3.8 (11) | 4.4 (6) | 2.3 (1) |
| Localization |  |  |  |
| *Femorotibial* | 69.1 (201) | 72.3 (99) | 77.3 (34) |
| *Patellofemoral* | 13.1 (38) | 13.9 (19) | 13.7 (6) |
| *FT + PF* | 14.4 (42) | 9.5 (13) | 4.5 (2) |
| *NA* | 3.4 (10) | 4.3 (6) | 4.5 (2) |
| Sport practice |  |  |  |
| *None* | 34.4 (100) | 39.4 (54) | 45.5 (20) |
| *Active* | 63.6 (185) | 60.6 (83) | 54.5 (24) |
| *NA* | 2.0 (6) |  | 0 (0) |
| Global health |  |  |  |
| *Poor* | - | 10.2 (14) | - |
| *Moderate* | - | 17.5 (24) | - |
| *Excellent* | - | 23.4 (32) | - |
| *NA* | - | 48.9 (67) | - |

Data represent frequency (number of patients); y : year; BMI : Body Mass Index; HA : hyaluronic acid; CTC : corticosteroids; PRP : platelets rich plasma; KL : Kellgren Laurence; FT : femorotibial; PF : patellofemoral; NA : not available

Table S2. Biological characterization of PRP obtained with Hy Tissue Tube 20 and Hy Tissue Tube 50.

|  | Hy Tissue Tube 20 (n=230) | Hy Tissue Tube 50 (n=201) |
| --- | --- | --- |
| Blood Sample |  |  |
| Net Volume harvested (mL) | 18 | 45 |
| Platelets Concentration (G/L) | 230 ± 56 | 225 ± 52 |
| PRP |  |  |
| Recovery Rate (%) | 83.5 ± 13.1 | 83.7 ± 12.3 |
| Volume injected (mL) | 8.6 ± 1.2 | 17.0 ± 3.1 |
| Platelets Concentration (G/L) | 430 ± 131 | 531 ± 137 |
| RBCs concentration (T/L) | 0.01 ± 0.01 | 0.02 ± 0.01 |
| Leukocytes concentration (G/L) | 0.26 ± 0.78 | 1.88 ± 1.51 |
| Platelets Dose (billion, x10^9^)  and proportion (%) | 3.7 ± 1.1  97.6 ± 1.7 | 9.0 ± 2.9  95.2 ± 2.2 |
| RBCs dose (million, x10^6^)  and proportion (%) | 89 ± 77  2.3 ± 1.9 | 348 ± 170  4.1 ± 1.9 |
| Leucocytes dose (million, x10^6^)  and proportion (%) | 2 ± 8  0.07 ± 0.21 | 38 ± 98  0.70 ± 3.30 |
| Increase factor in platelets | 1.9 ± 0.4 | 2.4 ± 0.4 |
| Increase factor in leukocytes | 0.04 ± 0.07 | 0.3 ± 0.3 |

Data represent mean ± standard deviation ; PRP : platelets rich plasma; RBCs : red blood cells

Table S3. Baseline Characteristics of patients treated with Hy Tissue Tube 20 that completed follow-up at 3 months (n= 230), 6 months (n=168), 12 months (88) and 18 months (n=35)

|  | Baseline & 3 months | 6 months | 12 months | 18 months |
| --- | --- | --- | --- | --- |
| Men | 46.5 (107) | 43.5 (73) | 37.5 (33) | 42.8 (15) |
| Age (y) |  |  |  |  |
| *18-39* | 6.5 (15) | 5.9 (10) | 3.4 (3) | 5.7 (2) |
| *40-59* | 37.8 (87) | 36.9 (62) | 38.6 (34) | 45.7 (16) |
| *60-79* | 48.7 (112) | 50 (84) | 56.8 (50) | 48.6 (17) |
| *≥80* | 7 (16) | 7.2 (12) | 1.2 (1) | 0 (0) |
| BMI (kg/m²) |  |  |  |  |
| *<25* | 34.8 (80) | 33.9 (57) | 35.2 (31) | 34.3 (12) |
| *25-30* | 39.6 (91) | 39.3 (66) | 43.2 (38) | 45.7 (16) |
| *>30* | 16.5 (38) | 19.1 (32) | 15.9 (14) | 20 (7) |
| *NA* | 9.1 (21) | 7.7 (13) | 5.7 (5) | 0 (0) |
| Previous treatment |  |  |  |  |
| *None* | 39.6 (91) | 39.3 (66) | 50 (44) | 51.4 (18) |
| *HA* | 35.6 (82) | 35.7 (60) | 25 (22) | 20 (7) |
| *CTC* | 12.2 (28) | 12.5 (21) | 13.6 (12) | 17.1 (6) |
| *PRP* | 7.8 (19) | 8.9 (15) | 10.2 (9) | 11.5 (4) |
| *≥ 2* | 3 (7) | 1.8 (3) | 0 (0) | 0 (0) |
| *NA* | 1.3 (3) | 1.8 (3) | 1.2 (1) | 0 (0) |
| KL grade |  |  |  |  |
| *1* | 18.3 (42) | 19 (32) | 22.7 (20) | 20 (7) |
| *2* | 35.6 (82) | 32.1 (54) | 28.4 (25) | 34.3 (12) |
| *3* | 25.2 (58) | 25.6 (43) | 25 (22) | 22.9 (8) |
| *4* | 16.9 (39) | 18.5 (31) | 18.2 (16) | 20 (7) |
| *NA* | 4 (9) | 5.8 (8) | 5.7 (5) | 2.8 (1) |
| Localization |  |  |  |  |
| *Femorotibial* | 60.4 (139) | 61.3 (103) | 65.9 (58) | 71.5 (25) |
| *Patellofemoral* | 14.8 (34) | 15.5 (26) | 14.8 (13) | 17.1 (6) |
| *FT + PF* | 20.9 (48) | 18.5 (31) | 13.6 (12) | 5.7 (2) |
| *NA* | 3.9 (9) | 4.7 (8) | 5.7 (5) | 5.7 (2) |
| Sport practice |  |  |  |  |
| *None* | 35.6 (82) | 35.7 (60) | 38.6 (34) | 48.6 (17) |
| *Active* | 63.5 (146) | 63.1 (106) | 61.4 (54) | 51.4 (18) |
| *NA* | 0.9 (2) | 1.2 (2) | 0 (0) | 0 (0) |
| Global health |  |  |  |  |
| *Poor* | 12.6 (29) | - | 21.6 (19) | - |
| *Moderate* | 25.3 (58) | - | 22.7 (20) | - |
| *Excellent* | 23.9 (55) | - | 10.2 (9) | - |
| *NA* | 38.2 (88) | - | 45.5 (40) | - |

Data represent frequency (number of patients); y : year; BMI : Body Mass Index; HA : hyaluronic acid; CTC : corticosteroids; PRP : platelets rich plasma; KL : Kellgren Laurence; FT : femorotibial; PF : patellofemoral; NA : not available

Table S4. Baseline Characteristics of patients treated with Hy Tissue Tube 50 that completed follow-up at 3 months (n= 201), 6 months (n=123), 12 months (49) and 18 months (n=9)

|  | Baseline & 3 months | 6 months | 12 months | 18 months |
| --- | --- | --- | --- | --- |
| Men | 55.2 (111) | 54.5 (67) | 44.9 (22) | 55.6 (5) |
| Age (y) |  |  |  |  |
| *18-39* | 2 (4) | 0 (0) | 0 (0) | 0 (0) |
| *40-59* | 35.3 (71) | 37.4 (46) | 38.8 (19) | 22.2 (2) |
| *60-79* | 52.8 (106) | 51.2 (63) | 53.1 (26) | 55.6 (5) |
| *≥80* | 9.9 (20) | 11.4 (14) | 8.1 (4) | 22.2 (2) |
| BMI (kg/m²) |  |  |  |  |
| *<25* | 37.3 (75) | 34.1 (42) | 34.7 (17) | 55.6 (5) |
| *25-30* | 42.8 (86) | 42.3 (52) | 46.9 (23) | 33.3 (3) |
| *>30* | 18.9 (38) | 22.8 (28) | 16.3 (8) | 11.1 (1) |
| *NA* | 1 (2) | 0.8 (1) | 2.1 (1) | 0 (0) |
| Previous treatment |  |  |  |  |
| *None* | 54.2 (109) | 58.6 (72) | 57.2 (28) | 88.9 (8) |
| *HA* | 21.4 (43) | 22.8 (28) | 22.4 (11) | 11.1 (1) |
| *CTC* | 9.5 (19) | 8.9 (11) | 10.2 (5) | 0 (0) |
| *PRP* | 11.9 (24) | 8.1 (10) | 10.2 (5) | 0 (0) |
| *≥ 2* | 2 (4) | 0.8 (1) | 0 (0) | 0 (0) |
| *NA* | 1 (2) | 0.8 (1) | 0 (0) | 0 (0) |
| KL grade |  |  |  |  |
| *1* | 7.5 (15) | 4.9 (6) | 4.1 (2) | 11.1 (1) |
| *2* | 37.3 (75) | 36.6 (45) | 48.9 (24) | 55.6 (5) |
| *3* | 32.3 (65) | 34.1 (42) | 32.7 (16) | 33.3 (3) |
| *4* | 20.9 (42) | 22 (27) | 12.2 (6) | 0 (0) |
| *NA* | 2 (4) | 2.4 (3) | 2.1 (1) | 0 (0) |
| Localization |  |  |  |  |
| *Femorotibial* | 78.1 (157) | 79.7 (98) | 85.7 (42) | 100 (9) |
| *Patellofemoral* | 8.5 (17) | 9.8 (12) | 12.2 (6) | 0 (0) |
| *FT + PF* | 11.9 (24) | 8.9 (11) | 2.1 (1) | 0 (0) |
| *NA* | 1.5 (3) | 1.6 (2) | 0 (0) | 0 (0) |
| Sport practice |  |  |  |  |
| *None* | 31.8 (64) | 32.5 (40) | 40.8 (20) | 66.7 (6) |
| *Active* | 64.7 (130) | 64.3 (79) | 59.2 (29) | 33.3 (3) |
| *NA* | 3.5 (7) | 3.2 (4) | 0 (0) | 0 (0) |
| Global health |  |  |  |  |
| *Poor* | 11.9 (24) | - | 10.2 (5) | - |
| *Moderate* | 24.9 (50) | - | 8.2 (4) | - |
| *Excellent* | 24.9 (50) | - | 26.5 (13) | - |
| *NA* | 38.3 (77) | - | 55.1 (27) | - |

Data represent frequency (number of patients); y : year; BMI : Body Mass Index; HA : hyaluronic acid; CTC : corticosteroids; PRP : platelets rich plasma; KL : Kellgren Laurence; FT : femorotibial; PF : patellofemoral; NA : not available

Table S5. Clinical outcomes for patients treated with Hy Tissue Tube 20

|  | M0 | M3 | M6 | M12 | M18 |
| --- | --- | --- | --- | --- | --- |
| Number of patients | 230 | 230 | 168 | 88 | 35 |
| WOMAC Score | 37.9 ± 18.5 | 27.5 ± 18.7 | 26.2 ± 19.7 | 26.4 ± 18.6 | 25.5 ± 16.9 |
| Change in  WOMAC score | - | -10.4 ± 16.4 | -11.0 ± 17.3 | -9.9 ± 17.4 | -13.6 ± 21.1 |
| Proportion (n) of patients improved | - | 75.2 (173) | 73.2 (123) | 70.4 (62) | 74.3 (26) |
| % improvement (improved patients) | - | 43.2 ± 27.4 | 50.6 ± 30.4 | 48.7 ± 30.1 | 45.6 ± 30.4 |
| % improvement  (total patients) | - | 15.5 ± 97.0 | 16 ± 125 | 22.4 ± 53.9 | 27.4 ± 41.5 |
| Pain VAS | 55.8 ± 19.1 | 36.8 ± 22.9 | 36.3 ± 23.4 | 36.9 ± 23 | 36.6 ± 23.9 |
| Change in  pain VAS | - | -19 ± 24.5 | -18.7 ± 25.3 | -18.1 ± 25.4 | -19.7 ± 30.6 |
| Proportion (n) of patients improved | - | 61.3 (141) | 69.6 (117) | 69.3 (61) | 71.4 (25) |
| % improvement (improved patients) | - | 48.8 ± 25.4 | 54.8 ± 26.6 | 54.9 ± 27.2 | 52.2 ± 30.3 |
| % improvement (total patients) | - | 27.0 ± 65.6 | 24.8 ± 83 | 25.1 ± 62.7 | 26.5 ± 52.3 |

Data represent mean ± standard deviation except for proportion of patients improved represented as frequency (number of patients).

Table S6. Clinical outcomes for patients treated with Hy Tissue Tube 50

|  | M0 | M3 | M6 | M12 | M18 |
| --- | --- | --- | --- | --- | --- |
| Number of patients | 201 | 201 | 123 | 49 | 9 |
| WOMAC Score | 41.8 ± 19.1 | 31.2 ± 19.7 | 31.5 ± 20.4 | 24.9 ± 21.8 | 15.2 ± 16.2 |
| Change in  WOMAC score | - | -10.6 ± 17.2 | -11.2 ± 17.5 | -13.8 ± 15.2 | -19.7 ± 21.7 |
| Proportion (n) of patients improved | - | 73.6 (145) | 76.4 (94) | 79.6 (39) | 55.6 (5) |
| % improvement (improved patients) | - | 44.1 ± 24.9 | 45.1 ± 26.1 | 54.7 ± 35.0 | 81.5 ± 25.8 |
| % improvement  (total patients) | - | 21.8 ± 49.2 | 21.3 ± 68.3 | 38.8 ± 46.3 | 53.0 ± 47.6 |
| Pain VAS | 56.3 ± 21.7 | 41.3 ± 23.6 | 40.9 ± 26.2 | 32.5 ± 26.1 | 20.0 ± 11.9 |
| Change in  pain VAS | - | -15.1 ± 21.7 | -18.5 ± 22.3 | -19.6 ± 24.7 | -13.8 ± 26.2 |
| Proportion (n) of patients improved | - | 67.2 (135) | 69.1 (85) | 69.4 (34) | 44.4 (4) |
| % improvement (improved patients) | - | 46.9 ± 25.3 | 52.3 ± 24.1 | 61.4 ± 27.7 | 68.2 ± 21.7 |
| % improvement (total patients) | - | 23.2 ± 57.7 | 30.7 ± 43.4 | 33.2 ± 60.8 | 3.3 ± 94 |

Data represent mean ± standard deviation except for proportion of patients improved represented as frequency (number of patients).
